# Extension of Japan’s Prefectural Emission Accounting and Enrichment of Socioeconomic Data from 1990 to 2020

**DOI:** 10.1038/s41597-024-03316-x

**Published:** 2024-05-11

**Authors:** Zhiheng Chen, Liqiao Huang, Yang Liu, Yoshida Yoshikuni, Kenji Tanaka, Yin Long

**Affiliations:** 1 Graduate School of Engineering, University of Tokyo, Tokyo, Japan; 2 School of International Trade and Economics, University of International Business and Economics, Beijing, 100029 China

**Keywords:** Energy efficiency, Energy and behaviour, Environmental impact, Energy policy

## Abstract

With the continuous increase in carbon dioxide emissions due to human activities and the resulting severe climate issues, there is global concern about energy conservation and emission reduction. However, detailed data on energy consumption and emissions at a fine-grained scale, particularly regarding spatial dimensions and sector-specific emissions, remains insufficient and in need of refinement and timely updates. In Japan, following the Fukushima nuclear disaster, there has been a significant shift from nuclear power generation to reliance on fossil fuels across various sectors, highlighting disparities in emissions data across different regions and industries. Our work extends the emissions time series for Japan’s 47 prefectures, incorporating their socioeconomic characteristics over a broader time frame and with a more detailed sectoral classification. The emissions inventory, covering the period from 1990 to 2020, is based on the consumption of the three main fossil fuels across 32 sectors, with emissions carefully allocated for regional power generation. This dataset, presented in a unified format, is expanded to include longer time scales and more detailed socioeconomic data. It is anticipated to offer crucial insights for establishing regional emission reduction targets and identifying sectoral priorities for decarbonization.

## Background & Summary

The persistent rise in global temperatures presents a complex challenge, directly contributing to a spectrum of climatic issues that threaten human survival. According to the Global Carbon Project’s 2023 report, global carbon emissions have reached an alarming level of approximately 40.9 billion tons^1^. A significant portion of these emissions, about 36.8 billion tons, originates from the combustion of fossil fuels^1^. Statistical data indicate that the global average temperature has risen by approximately 1.1 degrees Celsius (°C) since the Industrial Revolution^2^, and it is likely that global warming will exceed 1.5 °C before 2030 and may surpass 1.7 °C shortly thereafter^1^. The escalation of global temperatures has led to an increase in the frequency and intensity of extreme weather events, posing severe threats to global food and water security^3–10^, human health^11–16^, and the economic and social fabric^17–23^. The latest 28th Conference of the Parties (COP28) to the United Nations Framework Convention on Climate Change has conducted its first “Global Stocktake” to assess the progress of climate action since the signing of the Paris Agreement, underscoring an anticipated shortfall of 20.3 to 23.9 billion tonnes of CO_2_ equivalent in achieving the 2030 goals^24^. As the fifth-largest emitter, Japan’s annual fossil CO_2_ emissions in 2022 were 1075.07 million tons, accounting for 2.83% of the global total^1^. Japan’s efforts in emission reduction are of significant importance to the global response to climate change.

Japan’s carbon reduction efforts have yielded some results, with historical emissions since 1990 exhibiting a fluctuating decline across various sectors^25^. In 2020, Japan declared its intention to achieve carbon neutrality by the year 2050, a commitment that was enshrined in the Act on Promotion of Global Warming Countermeasures^26^. The subsequent year, Japan revised its Nationally Determined Contribution (NDC), elevating its reduction target from an initial 26% to a 46% cut by 2030 relative to 2013 emissions^27^. This revision is set to lower Japan’s absolute carbon emissions from 1.079 billion tons per year to 813 million tons per year. By the end of 2023, Japan had achieved approximately 20% of its emissions reduction target, yet the ambitious decarbonization goals still require more concerted efforts. In February 2023, the Japanese government passed the Green Transformation (GX) Basic Policy, aiming to strengthen decarbonization in key industrial sectors through the GX Alliance and to promote renewable energy as the main source of electricity^28^. Japan is also exploring positive measures, including the implementation of a carbon pricing policy based on the GX Promotion Act and halting the construction of new coal-fired power plants without reduction measures within the country^29,30^. Following the 2011 Fukushima nuclear disaster, Japan had once contemplated abandoning nuclear power development in favor of fossil fuels^31^. Recently, the government has been considering the restart of idle reactors and the construction of new ones^28^. While nuclear power may see a resurgence in Japan’s future, safety reviews, and political obstacles are likely to make it challenging for nuclear energy to contribute to Japan’s 2030 targets.

Carbon emissions inventories provide the mechanism for discerning comprehensive emission metrics and tracing source outlines within delineated geographic perimeters over established temporal spans^32,33^. The scientific rigor and precision of carbon emission inventories are critical in pinpointing emission sources, evaluating potential reduction measures, and shaping as well as appraising mitigation strategies^34^. Recent research has expanded the ambit of carbon footprint assessments, underscoring the significance of sector-specific analyses in domains such as households^35–39^, food systems^10,40,41^, and transportation sectors^42–44^. This underlines the need for quantifying carbon emissions with enhanced spatial and temporal resolution. With the improvement of fine-scale data and methodologies, attention is increasingly focused on compiling carbon emission inventories at the national^45–48^, regional^34,49–53^, and household^54–56^ levels, as well as within specific subdomains^57,58^. In service of decarbonization goals, there is a growing demand for more refined carbon emission inventories in future research^59^.

Japanese government agencies have not disclosed carbon emission data involving detailed regions and sectors^31^. To address this, Our previous work has developed an emissions inventory database that includes 47 prefectures and 26 sectors (including the power sector)^31^. Nonetheless, the database could be enhanced in two key areas: Firstly, it currently spans from 2007 to 2015, and incorporating the most recent energy consumption statistics from Japan warrants extending this timeframe to capture a more comprehensive temporal scope. Secondly, the granularity of the 26 sectors included in the database does not meet the nuanced requirements of future research; availability of updated data facilitates a more detailed sectoral breakdown.

To accommodate the emerging needs of future research, we have updated the dataset based on previous work. This dataset seeks to bridge the identified gaps by expanding the scope of Japan’s prefectural carbon emission inventory, both temporally and sectorally. The temporal coverage of the database has been expanded to encompass a broader timeframe, from 1990 to 2020, enabling a more extensive quantification of regional historical carbon emission fluctuations. Additionally, the sectors within the database have been further delineated, with the total number now increased to 32. This includes separating previously aggregated sectors and consolidating those that cannot be classified into a single category, as detailed in Table 1. Simultaneously, to match each emission result, we compiled a corresponding set of socioeconomic data, including population, Gross Domestic Product (GDP), land prices, green spaces, and roads. Consistent with the previous version, we continue to use data from Japanese power companies and power plants to calculate carbon emissions from the power sector, and additionally estimate carbon emissions from the use of coal, crude oil, and natural gas not utilized for power generation. Moreover, to eliminate the effects of price level fluctuations, the socioeconomic data set was converted to constant 2011 prices.

**Table 1:** Comparison between the updated and original sector classifications.

No.	Previous Classification	No.	Updated Classification
	Sector name		Sector name
1	Agriculture, Forestry and Fishery	1	Agriculture, Forestry and Fishery
2	Mining, Quarrying of Stone and Gravel	2	Mining, Quarrying of Stone and Gravel
3	Construction Work Industry	3	Construction Work Industry
4	Manufacture of Food, Beverages, Tobacco and Feed	4	Manufacture of Food, Beverages, Tobacco and Feed
5	Manufacture of Textile Mill Products	5	Manufacture of Textile Mill Products
6	Manufacture of Pulp, Paper and Paper Products	6	Manufacture of Pulp, Paper and Paper Products
7	Printing and Allied Industries	7	Printing and Allied Industries
8	Manufacture of Chemical and Allied Products, Oil and Coal Products Manufacture of Plastic Products, Rubber Products and Leather Products	8	Manufacture of Chemical and Allied Products, Oil and Coal Products
		9	Manufacture of Plastic Products, Rubber Products and Leather Products
9	Manufacture of Ceramic, Stone and Clay Products	10	Manufacture of Ceramic, Stone and Clay Products
10	Manufacture of Iron and Steel	11	Manufacture of Iron and Steel
11	Manufacture of Machinery	12	Manufacture of Machinery
12	Manufacture of Lumber, Wood Products, Furniture and Fixtures Miscellaneous Manufacturing Industry	13	Manufacture of Lumber, Wood Products, Furniture and Fixtures
		14	Miscellaneous Manufacturing Industry
13	Electricity, Gas, Heat Supply and Water	15	Electricity, Gas, Heat Supply and Water
14	Information and Communications	16	Information and Communications
15	Transportation and Postal Activities	17	Transport and Postal Activities
16	Wholesale and Retail Trade	18	Wholesale and Retail Trade
17	Finance and Insurance	19	Finance and Insurance
18	Real Estate and Goods Rental and Leasing	20	Real Estate and Goods Rental and Leasing
19	Scientific Research, Professional and Technical Services	21	Scientific Research, Professional and Technical Services
20	Accommodations, Eating and Drinking Services	22	Accommodations, Eating and Drinking Services
21	Education, Learning Support	23	Education, Learning Support
22	Medical, Health Care and Welfare	24	Medical, Health Care and Welfare
23	Living Related and Personal Services and Amusement Services Compound Services Miscellaneous Services	25	Living Related and Personal Services and Amusement Services
		26	Miscellaneous Services
		27	Compound Services
24	Government	28	Government
25	Residential	29	Residential
26	Non-Energy	30	Non-Energy
		31	Unable to Classify
		32	Transportation

## Methodology

### Sectoral emissions accounting

The scope of emission accounting includes Japan’s four major sectors: industrial sector, household sector, transportation sector and other non-energy sectors, totaling 32 sectors. The original data used for calculations come from energy consumption data from Japan’s prefecture-level natural resources and energy departments (URL: https://www.enecho.meti.go.jp/statistics/energy_consumption/ec002/results.html#headline4), including the consumption of fossil fuels such as coal, crude oil and natural gas in each department. However, there is a lack of three types of fuel consumption data for the power generation sector, so in addition to non-electricity fuel emissions, we calculated power generation emissions separately. Emissions from power generation are allocated through emissions from power plants and are not further divided by fuel type, but as a single emission source alongside the three major fuels. With the exception of the power generation sector, all other sectors are estimated using energy consumption data.

For the estimation of power generation emissions, we collected power generation data of major electric power companies in the target years from the Federation of Electric Power Companies of Japan (Fuel performance. URL: https://pdb.fepc.or.jp) and obtained capacity data for each power plant from Japan National Land Numerical Information (Category: Facilities. URL: https://nlftp.mlit.go.jp/ksj/gml/datalist/KsjTmplt-P03.html) for prefectural power generation emissions distribution. Regional power plants generally provide electricity for the local area and some surrounding cities, so the total emissions can be estimated by referring to the number and capacity scale of power companies in the prefecture. Combining the three fossil fuels, sectoral emissions of prefectures can be calculated by the following formula:

1 $$\begin{array}{c}E{P}_{pt}=CA{P}_{pt}\times \sum _{i=1}\left({L}_{\theta pt}^{i}\times {H}_{it}\times {I}_{it}\right)\end{array}$$

2 $$\begin{array}{c}{E}_{ijpt}={G}_{ijpt}\times {H}_{it}\times {I}_{it}\end{array}$$

3 $$\begin{array}{c}{E}_{pt}=\sum _{i=1}\sum _{j=1}{E}_{ijpt}+E{P}_{pt}\end{array}$$

Where *EP*_*pt*_ is the power generation emissions in prefecture *p* in year *t*. *CAP*_*pt*_ is the proportion of power plant installed capacity of prefecture *p* in the area where it is supported by power company *θ*. This parameter comes from Japan National Land Numerical Information (Category: Facilities). ${L}_{\theta pt}^{i}$ represents the total consumption of fuel type *i* in year *t* by the power company *θ* that provides power support for prefecture *p*. This parameter comes from Electricity Statistics Information of The Federation of Electric Power Companies of Japan (FEPC). *H*_*it*_ represents the calorific value generated per unit of fuel type *i* consumed in year *t*, and *I*_*it*_ represents the corresponding emission intensity. These two values come from the Prefecture Energy Statistics of Agency for Natural Resources and Energy of Japan, as shown in Table 2. The calorific value and emission intensity coefficient of each fuel type are given by year. Therefore, ${\sum }_{i=1}\left({L}_{\theta pt}^{i}\times {H}_{it}\times {I}_{it}\right)$ can be understood as the total emissions from power generation using three fossil fuels in each prefecture. In Eq. (2), *E*_*ijpt*_ represents the total carbon dioxide emissions produced by sector *j* in prefecture *p* in year *t* consuming fuel type *i*. *G*_*ijpt*_ represents the consumption of fuel type *i* in sector *j* in prefecture *p* in year *t*. The data is driven by prefectural energy statistics from the Prefecture Energy Statistics of Agency for Natural Resources and Energy of Japan. In Eq. (3), *E*_*pt*_ represents the total emissions of prefecture *p* in year *t*.

**Table 2:** Fuel types and corresponding caloric value by year.

Fuel types of this study (i)	Fuels in Japan Prefecture Energy Statistics	Unit	Year (t)	*H*_*i*_	*I*_*i*_
				*TJ*	*ton of carbon*
				*Measuring Unit*	*TJ*
Coal		10^3^ t	1990	26.0	23.65
			2005	25.7	24.51
			2007	25.7	24.51
			2008	25.7	24.51
			2009	25.7	24.51
			2010	25.7	24.51
			2011	25.7	24.51
			2012	25.7	24.51
			2013	26.0	24.53
			2014	26.0	24.53
			2015	26.0	24.53
			2016	26.0	24.53
			2017	26.0	24.53
			2018	26.1	24.60
			2019	26.1	24.60
			2020	26.1	24.60
Oil		10^3^ kl	1990	38.3	18.66
			2005	38.1	18.66
			2007	38.1	18.66
			2008	38.1	18.66
			2009	38.1	18.66
			2010	38.2	18.66
			2011	38.2	18.66
			2012	38.1	18.66
			2013	38.2	19.00
			2014	38.2	19.00
			2015	38.2	19.00
			2016	38.2	19.00
			2017	38.2	19.00
			2018	38.2	18.98
			2019	38.1	18.98
			2020	38.1	18.98
Gas	Natural Gas	10^3^ t	1990	54.5	13.47
			2005	54.5	13.47
			2007	54.5	13.47
			2008	54.5	13.47
			2009	54.5	13.47
			2010	54.5	13.47
			2011	54.5	13.47
			2012	54.5	13.47
			2013	54.5	13.95
			2014	54.5	13.95
			2015	54.5	13.95
			2016	54.5	13.95
			2017	54.5	13.95
			2018	54.7	13.87
			2019	54.7	13.87
			2020	54.7	13.87
	Town Gas	10^6^ Nm^3^	1990	45.7	13.94
			2005	48.9	13.94
			2007	48.9	13.94
			2008	48.9	13.94
			2009	48.9	13.94
			2010	48.9	13.94
			2011	48.9	13.94
			2012	48.9	13.94
			2013	44.5	14.04
			2014	44.5	14.04
			2015	44.4	14.04
			2016	44.4	14.04
			2017	44.5	14.04
			2018	43.6	13.95
			2019	43.6	13.95
			2020	43.6	13.95

### Sectoral consumption on electricity and its allocation

As mentioned above, the absence of precise data on fossil fuel consumption at the prefectural level within the power generation sector necessitates an estimation of emissions based on the distribution of electricity consumption. We first gathered data on electricity usage from ten prominent power companies (see power_company_cover_region.xlsx in the Excel folder in figshare) over a specified timeframe. Subsequently, emissions were reassigned according to the respective capacities of local power plants in each prefecture, contributing to the overall emissions calculation at the prefectural level. In cases where multiple power companies support a single prefecture, we designate the company with the broadest coverage as the primary contributor. Given that the most recent power generation data is available only up to 2015, we extrapolate and utilize the same statistical values from that year for subsequent years.

### Socioeconomic data

The socio-economic characteristics of cities are often used as input variables in carbon emission-driven models. Multivariate regression modeling between socioeconomic and carbon emissions data can help scholars understand what characteristics influence carbon emissions and to what extent, and how this influence varies across geographies and sectors. Therefore, to match each emission result, we additionally transformed the corresponding socioeconomic data set, including population, GDP, land prices, green space, and roads. The population data of each prefecture from 1990 to 2020 comes from the Statistics Bureau of Japan (Census. URL: https://www.e-stat.go.jp/). Prefectural GDP data comes from the Prefectural Final Accounts Annual Report of the Cabinet Office of Japan (URL: https://www.cao.go.jp/). Land price, green space and road data come from the Japan Land Numerical Information website (URL: https://nlftp.mlit.go.jp/ksj/index.html). Socio-economic indicators are recorded as follows:

1. Population (unit: person)
2. GDP (unit: million yen)
3. Average land price (unit: yen per square meter)
4. Per capita green space area (unit: square meters per person)
5. Road density (meters per square kilometer)

## Data Records

**Fig. 1:**
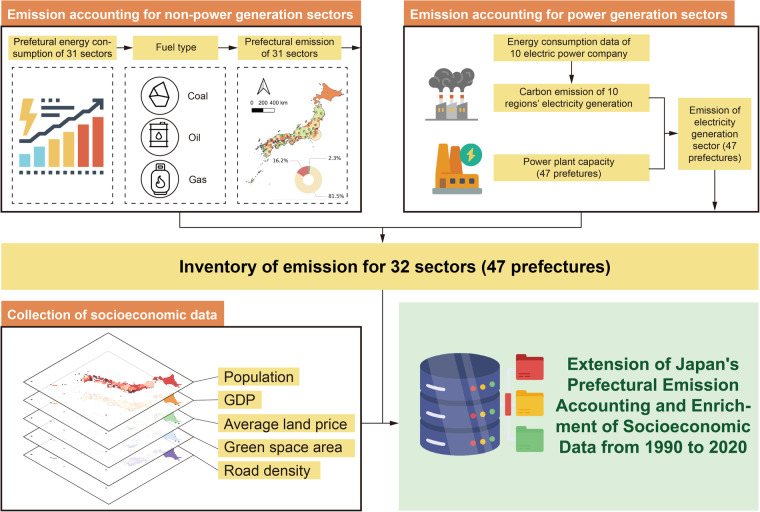
Flowchart of this dataset.

This dataset encompasses a comprehensive compilation of 72,192 data records, spanning 47 prefectures and 32 sectors (as delineated in Table 3), capturing energy consumption based on three types of fossil fuels and one secondary energy source (electricity) over 16 years (Data for 1991–2004 and 2006 are not collected in this dataset). The dataset, denominated “Extension of Japan Prefectural Emission Accounting and Enrich Socioeconomic Data 1990 to 2020,” is publicly accessible via Figshare^60^. To provide a detailed breakdown, the database is structured into seven main components. Firstly, it comprises an Excel folder containing data on prefecture-level heat generation, emissions, population, GDP, land prices, park area per capita, and road density in Japan from 1990 to 2020, offering valuable insights into energy consumption, economic indicators, and environmental factors. Secondly, six distinct folders house a historical dataset on prefecture-level heat generation and emissions categorized by energy sources and industries in Japan, spanning from 1990 to 2020. These folders also encompass metrics for heat generation and emissions normalized by GDP and per capita, presented as ‘.shp’ files, specifically designated as ‘Heat’, ‘Heat per capita’, ‘Heat per GDP’, ‘Emission’, ‘Emission per capita’, and ‘Emission per GDP’ respectively. Furthermore, the dataset incorporates a comprehensive socioeconomic inventory, featuring a substantial number of records, which comprehensively covers five major indicators. The flowchart of this dataset is shown as Fig. 1.

**Table 3:** Database Structure and Description.

Filename	Description
*Excel file*	
Heat.xlsx	Prefecture-level heat generated by energy sources by industries from 1990 to 2020
Heat_per_gdp.xlsx	Prefecture-level heat generated by energy sources by industries for per unit of GDP from 1990 to 2020
Heat_per_capita.xlsx	Prefecture-level heat generated by energy sources by industries for per capita from 1990 to 2020
Emission.xlsx	Prefecture-level emission generated by energy sources by industries from 1990 to 2020
Emission_per_gdp.xlsx	Prefecture-level emission generated by energy sources by industries for per unit of GDP from 1990 to 2020
Emission_per_capita.xlsx	Prefecture-level emission generated by energy sources by industries for per capita from 1990 to 2020
1990-2020JPpopulation.xlsx	Prefecture-level population from 1990 to 2020
1990-2019JPgdp.xlsx	Prefecture-level GDP by industries from 1990 to 2019
1983-2023JPPrefectureLandprice.xlsx	Prefecture-level average landprice from 1983 to 2023
1990-2020JPparkareapercapita.xlsx	Prefecture-level park area per capita from 1990 to 2020
2007-2020JProad.xlsx	Prefecture-level road density from 2007 to 2020
power_company_cover_region.xlsx	Ten major electric utilities and their service areas
*Shapefile*	
1990-2020JPpopulation.shp	Prefecture-level population from 1990 to 2020
1990-2019JPgdp.shp	Prefecture-level GDP by industries from 1990 to 2019
1983-2023JPPrefectureLandprice.shp	Prefecture-level average landprice from 1983 to 2023
1990-2020JPparkareapercapita.shp	Prefecture-level park area per capita from 1990 to 2020
2007-2020JProad.shp	Prefecture-level road density from 2007 to 2020
Heat_90FY.shp	Prefecture-level heat generated by energy sources by industries of 1990
…	…
Heat_20FY.shp	Prefecture-level heat generated by energy sources by industries of 2020
Heat_per_gdp_90FY.shp	Prefecture-level heat generated by energy sources by industries for per unit of GDP of 1990
…	…
Heat_per_gdp_20FY.shp	Prefecture-level heat generated by energy sources by industries for per unit of GDP of 2020
Heat_per_capita_90FY.shp	Prefecture-level heat generated by energy sources by industries for per capita of 1990
…	…
Heat_per_capita_20FY.shp	Prefecture-level heat generated by energy sources by industries for per capita of 2020
Emission_90FY.shp	Prefecture-level emission generated by energy sources by industries of 1990
…	…
Emission_20FY.shp	Prefecture-level emission generated by energy sources by industries of 2020
Emission_per_gdp_90FY.shp	Prefecture-level emission generated by energy sources by industries for per unit of GDP of 1990
…	…
Emission_per_gdp_20FY.shp	Prefecture-level emission generated by energy sources by industries for per unit of GDP of 2020
Emission_per_capita_90FY.shp	Prefecture-level emission generated by energy sources by industries for per capita of 1990
…	…
Emission_per_capita_20FY.shp	Prefecture-level emission generated by energy sources by industries for per capita of 2020

## Technical Validation

### Total sectoral emission by years

**Fig. 2:**
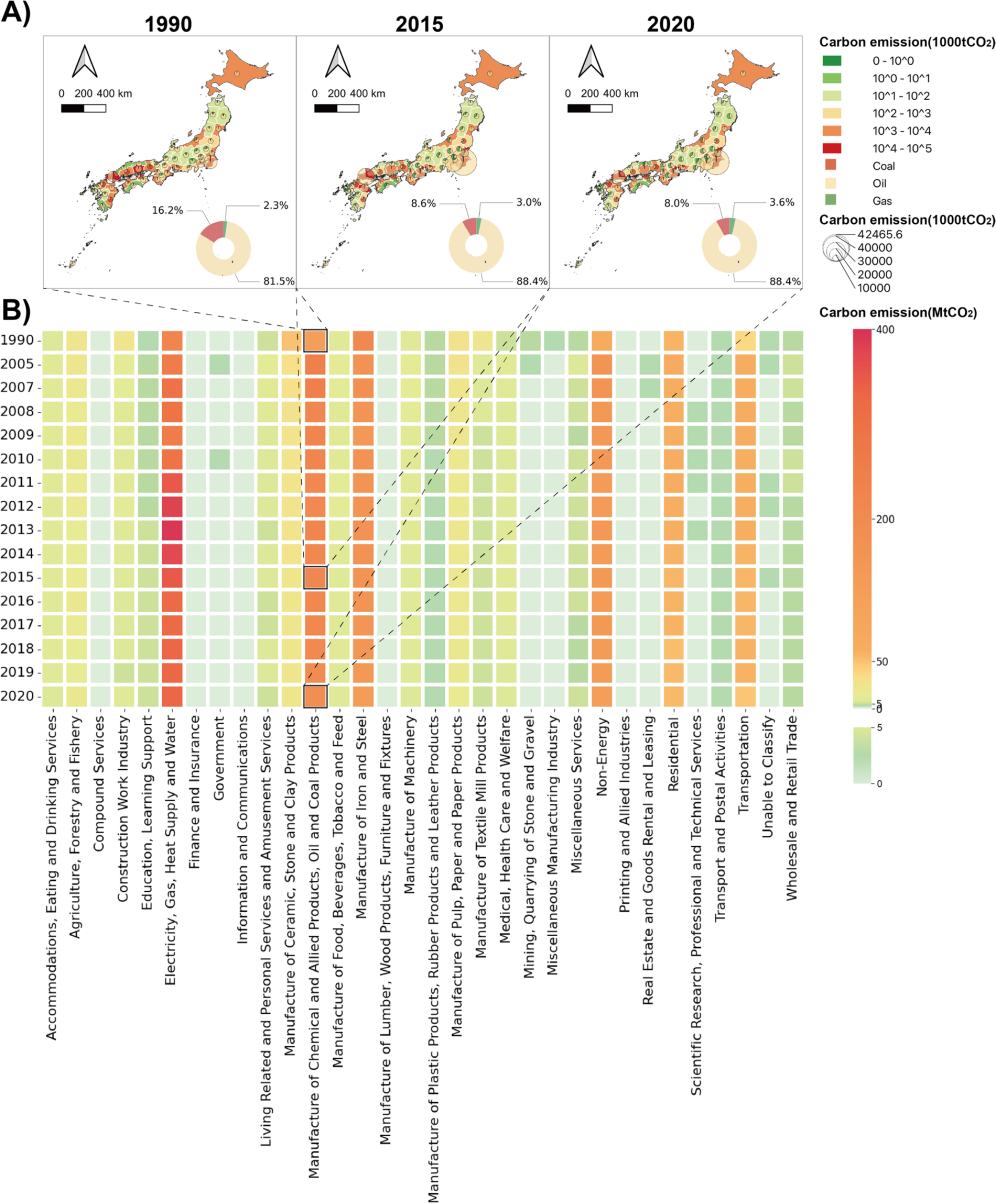
(**A**) Prefectural carbon dioxide emission of “Manufacture of Chemical and Allied Products, Oil and Coal Products” sector (unit: 1000tCO_2_). The donut pie chart represents the national share of emissions from the sector’s use of three fossil fuels. The pie chart on the map represents the share of emissions from each prefecture’s use of the three fossil fuels in that sector. The size of the pie chart represents the amount of emissions from that sector. (**B**) Sectoral carbon dioxide emission from 1990 to 2020 (unit: MtCO_2_).

Annual emissions by sector from 1990 to 2020 are given in Fig. 2(B). In addition to the power generation sector, the five sectors with the highest annual emissions in the observed years are “Manufacture of Chemical and Allied Products, Oil and Coal Products”, “Manufacture of Iron and Steel”, “Non-Energy”, “Residential” and “Transportation”. Fig. 2(A) provides a further understanding of emissions from the “Manufacturing of Chemical and Allied Products, Oil and Coal Products” sector in three years: 1990, 2015 and 2020, including the proportion of carbon emissions caused by fuel use nationwide, the emission and emission proportions of the three types of fuels in each region. The results show that from 1990 to 2015, the sector’s share of national emissions caused by burning coal decreased by 7.6%, while the share of oil and gas increased by 6.9% and 0.7% respectively. The proportion of oil has almost remained unchanged from 2015 to 2020, and a small amount (0.6%) of carbon emissions has shifted from coal to gas. The sector in central Japan has gradually shed its reliance on coal and oil over time and was dominated by natural gas by 2020 Fig. 2.

### Comparison with other estimation results

**Fig. 3:**
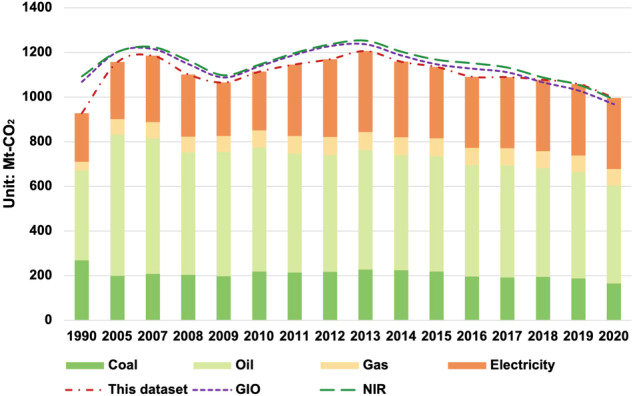
Comparison with other estimation results.

This data set compiles Japan’s prefecture-level emissions inventory based on energy consumption by sector. Here we compare the estimation results with The GHG Emission Data of Japan (URL: http://www.nies.go.jp/gio/en/archive/index.html) provided by the Greenhouse Gas Inventory Office of Japan (GIO) and the National Greenhouse Gas Inventory Report of Japan (NIR) released in April 2022 (URL: https://www.nies.go.jp/gio/en/archive/nir/index.html) (Fig. 3). The differences were largest in 1990, where the GIO difference ratio (assessment gap/GIO value) and NIR difference ratio (assessment gap/NIR value) were 13.2% and 15.2% respectively. The reason for the assessment gap may be changes in departmental classification. Japan’s Standard Industrial Classification was revised twice in 1993 and 2002, resulting in some newly added departments not being included in the 1990 energy consumption accounting. The biggest difference between the estimates after 2005 and those from other national estimates appeared around 2011. For example, the GIO difference ratios are 3.5% (2011) and 4.7% (2012), and the NIR difference ratios are 4.2% (2011) and 5.4% (2012). The difference in assessment mainly occurs in the assessment of power generation, because part of the power generation burden caused by the shutdown of nuclear power plants after the Great East Japan Earthquake in 2011 was transferred to private power generation (non-utility power generation facilities). The power generation and fuel consumption of these private power generation facilities are difficult to accurately survey and quantify. Therefore, the power generation estimates in this data set do not include private power generation, resulting in a gap between around 2011 and other assessment results. After 2015, the estimates from this data set gradually approached and exceeded other national estimates. The reason is that due to the lack of power generation data from 2016 to 2020, this study uses the same baseline value as in 2015 for power generation estimates in years after 2015. This may lead to overestimation of emissions from the power generation sector.

## Usage Note

The dataset is a pivotal resource for a variety of applications in environmental and socio-economic research. This extensive dataset, encompassing 96,256 records across 47 prefectures and 32 sectors, provides a detailed view of Japan’s carbon emissions and socioeconomic parameters over time. This compilation is derived from a meticulous amalgamation of data from multiple sources, including the electricity consumption dataset and publicly available data from the Japanese Government Statistics. Researchers and experts are afforded the flexibility to independently extract and process information from any database, adhering to our outlined methodology for data cleaning and enhancement.

As for the potential usage, policymakers and environmental planners can utilize this dataset to assess the impact of energy consumption in different sectors and prefectures on carbon emissions, which helps to inform targeted and effective environmental policies and initiatives at both local and national levels. Moreover, our dataset provides insights into the consumption patterns of fossil fuels and electricity across various sectors. Energy companies and consultants can analyze this data to identify trends and make informed decisions regarding energy production and distribution. The dataset’s strength lies in its detailed sectoral and regional breakdown, which, when used cautiously, can yield highly valuable insights for a wide range of applications. This dataset is openly accessible to the public, subject to the terms of the Creative Commons License with attribution (CC-BY 4.0).

## Data Availability

The code used for analysis in this study is publicly available at: https://github.com/chenzhiheng970717/SD_code.git.
